# Survival Outcome of Thoraco‐Laparoscopic McKeown Esophagectomy Versus Endoscopic Submucosal Dissection for Early‐Stage Esophageal Squamous Cell Carcinoma: A Propensity Score‐Matched Analysis

**DOI:** 10.1111/1759-7714.70064

**Published:** 2025-05-05

**Authors:** Ping Yuan, Zhenhao Huang, Feichao Bao, Jiajing Li, Lidong Chen, Hongtao Wen, Donglei Liu, Feng Li, Shanfeng Zhang, Yu Qi, Xiangnan Li

**Affiliations:** ^1^ Department of Thoracic Surgery The First Affiliated Hospital of Zhengzhou University Zhengzhou Henan China; ^2^ Department of Thoracic Surgery Shanghai Chest Hospital, Shanghai Jiao Tong University Shanghai China; ^3^ Department of Pathology The First Affiliated Hospital of Zhengzhou University Zhengzhou Henan China; ^4^ Department of Gastroenterology The First Affiliated Hospital of Zhengzhou University Zhengzhou Henan China; ^5^ School of Basic Medical Science Zhengzhou University Zhengzhou Henan China

**Keywords:** endoscopic submucosal dissection, esophageal squamous cell carcinoma, propensity score‐matched analysis, survival outcome, thoraco‐laparoscopic McKeown esophagectomy

## Abstract

**Objective:**

To evaluate thoraco‐laparoscopic McKeown esophagectomy (TLME) versus endoscopic submucosal dissection (ESD) for clinical‐T1N0 esophageal squamous cell carcinoma (ESCC) depending on invasion depth.

**Background:**

Early‐stage ESCC has been widely treated by endoscopic resection. While ESD is safer than esophagectomy perioperatively, its survival benefits for clinical‐T1N0M0 ESCC, especially high‐risk T1b tumors, are unclear.

**Methods:**

A retrospective study was conducted on clinical‐T1N0 ESCC patients at the First Affiliated Hospital of Zhengzhou University comparing TLME (cT1a, *n* = 352; cT1b, *n* = 205) with ESD (cT1a, *n* = 499; cT1b, *n* = 62). Overall survival (OS), disease‐specific survival (DSS), relapse‐free survival (RFS), and metastasis‐free survival (MFS) were analyzed depending on invasion depth after propensity score matching to account for selection bias.

**Results:**

ESD group had better OS (hazard ratio: 0.54, *p* = 0.029) but worse RFS (hazard ratio: 6.83, *p <* 0.001) than TLME group in general terms. T1a cancers showed no difference in DSS and MFS between groups. T1b subgroup with ESD had lower DSS (hazard ratio: 5.65, *p* = 0.036) and MFS (hazard ratio: 3.54, *p* = 0.069). R1‐resection in ESD group linked to poorer OS (hazard ratio: 5.89, *p* = 0.006) and DSS (hazard ratio: 3.67, *p* = 0.006).

**Conclusion:**

ESD can be safe in the treatment of clinical‐T1aN0 ESCC. However, concerning oncologic curability, TLME should be recommended for patients with clinical‐T1bN0 ESCC in terms of favorable DSS, RFS, and MFS.

## Graphical abstract

A image summarizing the survival analysis of patients in the ESD group and the TLME group after propensity score matching. For clinical‐T1aN0 ESCC, endoscopic submucosal dissection (ESD) demonstrated superior overall survival (OS) with comparable safety to thoraco‐laparoscopic McKeown esophagectomy (TLME). However, TLME provided significantly better disease‐specific survival (DSS) and relapse‐free survival (RFS) for T1b tumors, particularly in reducing metastasis risk. Propensity score‐matched analysis highlights TLME as the optimal curative strategy for high‐risk T1b ESCC, while ESD remains suitable for T1a lesions if R1‐resection is avoided.

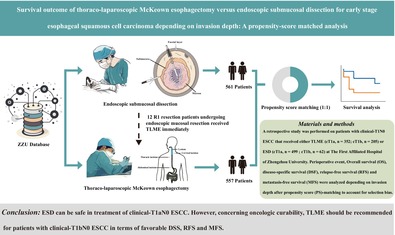

**Abbreviations**
ASD: absolute standardized mean difference
CRT: chemoradiotherapy
DSS: disease‐specific survival
EC: esophageal carcinoma
ER: endoscopic resection
ESCC: esophageal squamous cell carcinoma
ESD: endoscopic submucosal dissection
EUS: endoscopic ultrasonography
MFS: metastasis‐free survival
OS: overall survival
PS: propensity score
QOL: quality of life
RFS: relapse‐free survival
TLME: thoraco‐laparoscopic McKeown esophagectomy

## 1 Introduction

Esophageal carcinoma (EC) is the sixth leading cause of cancer deaths globally [1]. In China, over 90% of EC cases are esophageal squamous cell carcinoma (ESCC) [2, 3], which is more aggressive and has a poorer prognosis than esophageal adenocarcinoma [4]. However, early‐stage detection and surgical intervention can improve prognosis, with a 5‐year survival rate of 90% [5, 6].

Over the past decade, T1 stage ESCC within the mucous layer without lymph node metastasis (LNM) has increasingly been treated by endoscopic resection (ER) rather than esophagectomy [7]. Endoscopic submucosal dissection (ESD) has the greatest therapeutic potential among other ER procedures when R0‐resection is achieved [8]. However, its long‐term oncology safety is still undefined concerning untreated malignant lymph nodes [9, 10], particularly in T1b cancer that invades submucosa, which is believed to have a higher LNM risk [11, 12].

Thoraco‐laparoscopic McKeown esophagectomy (TLME) with lymph node excision is the most common surgical procedure for ESCC in educational hospitals across China [13]. It reduces morbidity and speeds up recovery compared to open procedures [14, 15]. Real‐world comparisons of ESD and TLME for ESCC are lacking. This study aimed to compare the oncology safety and survival of ESD and TLME for cT1N0 ESCC based on invasion depth.

## 2 Materials and Methods

### 2.1 Study Design and Population

This retrospective study used a prospectively assembled database (https://zzutsd.com) and institutional medical records. Patients who underwent esophagectomy and ER for clinical‐T1N0 ESCC between August 2012 and May 2022 were identified. Inclusion criteria were as follows: (1) underwent TLME or ESD, (2) T1 stage ESCC without LNMs based on clinic staging and pathology, (3) follow‐up ≥ 6 months. The number of ethics committee evaluation was 2021‐KY‐0388, which began on 5 October, 2021.

Image‐enhanced gastroscopy and biopsy were performed for the diagnosis of all patients. Endoscopic ultrasonography (EUS) was initially used to designate tumor depth. T1 stage was classified as either T1a (limited to muscularis mucosae) or T1b (invading submucosa). T1a lesions were further classified as T1a‐m1 (invading intra‐epithelium), T1a‐m2 (invading laminae propria mucosae), and T1a‐m3 (invading muscularis mucosa). Clinical lymph node examination included enhanced‐computerized tomography (CT) scans and positron emission tomography (PET)‐CT. For patients ready for ESD treatment, suspicious lymph node status was further examined by EUS fine‐needle aspiration or cervical ultrasound‐guided biopsy.

Patients who were initially diagnosed with superficial ESCC were scheduled for consultation from an institutional multi‐disciplinary team specialized in esophageal cancer, consisting of a pathologist, a radiologist, a gastroenterologist/endoscopist, and a thoracic surgeon. The curability and necessity of ESD were initially assessed, focusing on lymph node status and tolerance of general anesthesia and esophagectomy. Generally, patients with cT1a‐m1–cT1a‐m3 ESCC (confined to muscularis mucosa) without evidence of metastasis (cN0M0) were referred to the gastroenterology department. An endoscopic procedure was scheduled to attempt to eradicate the tumor by ESD. The margin and resectability were constantly evaluated by the endoscopist, who was ready to call off and transfer to the department of thoracic surgery. Patients with lesions invading the submucosa (cT1bN0M0) were referred to the department of thoracic surgery for esophagostomy. The final decision was determined by the patients and their families after being informed of the risks and benefits, and a written informed consent was signed.

### 2.2 TLME Techniques

MIME with cervical anastomosis was performed by two surgeons (Y.Q. and X.L.). Patients were under general anesthesia. The procedure began with four‐portal thoracoscopic esophageal and four‐port laparoscopic gastric mobilization. The sub‐xiphoid port was extended to a 5‐cm incision for gastric conduit formation and pulling. Mechanical cervical anastomosis was done with a circular stapler (CDH25, Ethicon TM), reinforced by circular sutures. A nasojejunal feeding tube was placed. Routine CT scans were done on the fourth day post‐surgery before nasojejunal feeding to check for surgical‐related issues. If the following signs, including extraluminal leakage at the anastomotic site, fluid collection, and gas bubbles in the mediastinum and pleural cavity, were detected, along with increased exudation from the neck dressing, fever, elevated white blood cell count, and increased inflammatory markers. Anastomotic leakage was highly suspected and confirmed by esophagography using a water‐soluble contrast agent [16]. After 2 days of nasojejunal feeding, patients were discharged. TLME patients were banned from oral feeding for 3 weeks, then readmitted for 2 days to attempt oral feeding and remove the tube. Enhanced CT scans were scheduled at 1 and 4 months post‐surgery, then every 6 months for 2 years, and annually. Digestive tract radiography was scheduled at 4 months, then every 6 months for 2 years, and annually. If the enhanced CT showed signal enhancement or the patient's symptoms were severe, or the patient exhibited relevant findings of ESCC on digestive tract radiography, an endoscopic examination would be performed.

### 2.3 ESD Techniques and Follow‐Up

ESD was performed by one endoscopist (H.W.) under general anesthesia. The dual‐knife and T‐knife were used. Marking dots were made 0.5 cm around the lesion. After submucosa separation by glycerol injection, a circular mucosal incision was made, and submucosal tissue was dissected from the muscularis propria. Hemostasis was controlled by coagulation or titanium clips. The lesion and margin were assessed throughout. ESD patients were banned from oral feeding until a barium radiograph at 3 days post‐ESD excluded esophageal fistula. Discharge was arranged after 2 days of oral feeding. Endoscopic surveillance with biopsies and enhanced CT scans was scheduled every 3 months for 1 year, every 6 months for 1–2 years, and annually thereafter.

### 2.4 Propensity Score Matching Analysis

Propensity score (PS) matching was performed on the entire sample and T stage subgroups using one‐to‐one nearest‐neighbor matching within a 0.05 standard deviation caliper. PS was estimated with gender, age, smoking and alcohol history, BMI, lesion diameter, ASA score, age‐adjusted Charlson comorbidity index (CCI), differentiation level, and LVI/PNI status, which were widely recognized to influence survival outcomes. Absolute standardized mean difference (ASD) was calculated to compare variable balance before and after PS matching, with ASD < 0.1 indicating negligible differences.

### 2.5 Survival Analysis

Overall survival (OS) was estimated using Kaplan–Meier (K–M) curves. The log‐rank test and Cox proportional hazards model were used to calculate the *p* value between two KM curves and the hazard ratio (HR) with its 95% confidence interval (CI), respectively. Considering that a small proportion of patients died from causes unrelated to ESCC before reaching the endpoint event, this could introduce a competing risk factor for disease‐specific survival (DSS), relapse‐free survival (RFS), and metastasis‐free survival (MFS). Therefore, in our analysis of DSS, RFS, and MFS, we used the Aalen‐Johansen estimator (lifelines library version 0.27.0) to calculate the cumulative incidence function (CIF) for each group and plotted the CIF curves. Additionally, we applied the Fine and Gray model (cmprsk package version 2.2.12) to compare the groups, calculating the *p* values, HR, and 95% CI. Analyses were performed using IBM SPSS v24.0, R (version 4.3.2) and Python (version 3.7.12). Survival curves were generated using GraphPad Prism v6.0 and Python (version 3.7.12). *p* < 0.05 were considered statistically significant. We conducted a subgroup analysis based on the depth of tumor infiltration and found that ESD can be safe in the treatment of clinical‐T1aN0 ESCC and lead to better OS, while TLME should be recommended for patients with clinical‐T1bN0 ESCC in terms of favorable DSS, RFS, and MFS when concerning oncologic curability.

### 2.6 Statistical Analysis

Categorical variables were compared using the chi‐squared test before PS‐matching and using the McNemar test after PS‐matching. Continuous variables were represented as mean with standard deviation. The normal or non‐normal distribution of continuous variables was determined by the Kolmogorov–Smirnov test. The homogeneity of continuous variance was tested by the Levene test. For continuous variables that followed a normal distribution, the Student's *t*‐test was used before PS‐matching and the paired *t*‐test was used after PS‐matching. For continuous variables that followed a non‐normal distribution, the paired *t*‐test was used before PS‐matching and the Wilcoxon signed‐rank test was used after PS‐matching.

## 3 Results

### 3.1 Patient Characteristics

**FIGURE 1:**
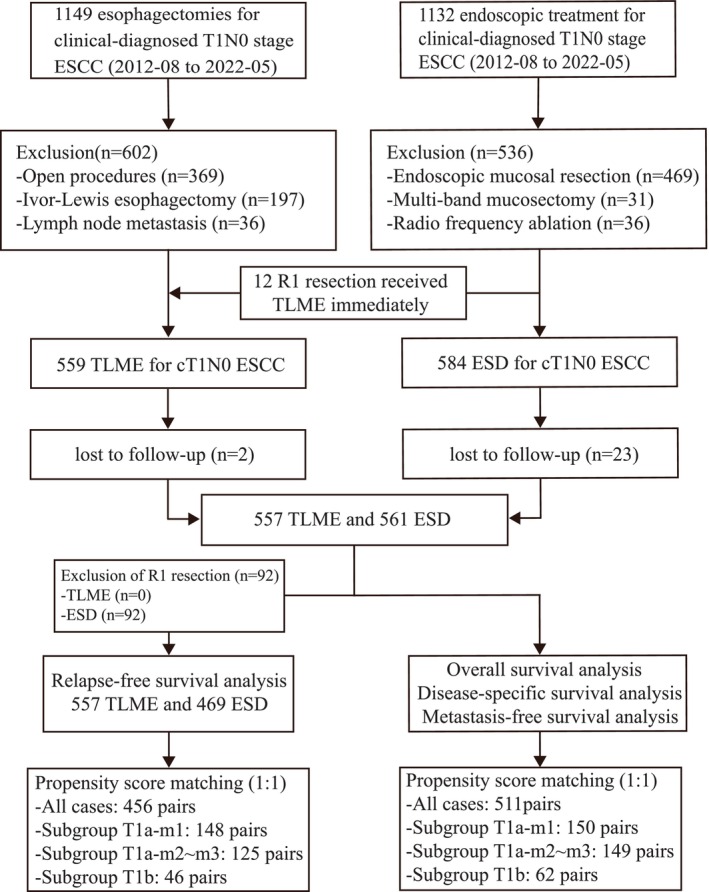
Flowchart depicting the basic dataset, exclusion criteria, and result of PS‐matching.

Data from 2281 patients who underwent esophagectomy or ER for cT1N0 ESCC from August 2012 to May 2022 were analyzed. For the TLME group, we excluded patients who received open procedures (*n* = 369), Ivor‐Lewis esophagectomies (*n* = 197), or lymph node cancer infiltration (*n* = 36). For the ESD group, exclusions included those who received endoscopic mucosal resection (*n* = 469), multi‐band mucosectomy (*n* = 31), or radiofrequency ablation (*n* = 36). Twelve patients initially treated with ESD moved to the TLME group due to R1‐resection, with TLME rescheduled 2 weeks post‐ESD. Twenty‐five patients lost to follow‐up were also excluded. After exclusions (Figure 1), the study included 557 patients in the TLME group and 561 in the ESD group.

Demographic and clinical characteristics are shown in Table 1, Table S1, and Table S4. Before PS‐matching, covariates were imbalanced (ASD > 0.10). PS‐matching generated 511 pairs, balancing all factors except T stage. Subgroup PS‐matching by T stage produced 150 pairs of T1a‐m1, 149 pairs of T1a‐m2–m3, and 62 pairs of T1b cancers. PS‐matching was also done for patients with R0 resection for RFS analysis. ASD for covariates before and after PS‐matching was shown in the covariance balance plot (Figure S1). All factors were well balanced (ASD < 0.10). Density plots were drawn to compare the overlap before PS‐matching and after PS‐matching (Figure S4).

**TABLE 1:** Demographic and clinical characteristics of all patients with calculated absolute standardized difference (ASD) before and after propensity score matching (PS‐matching).

Factor (no. %)	All patients		ASD	PS‐matched patients		ASD
	557 TLME	561 ESD		511 TLME	511 ESD	
Gender						
Male	341 (61.2)	320 (57.0)	0.08	304 (59.5)	308 (60.3)	0.01
Female	216 (38.8)	241 (43.0)	0.08	207 (40.5)	203 (39.7)	0.01
Age						
> 68 years	115 (20.6)	154 (27.5)	0.15	114 (22.3)	128 (25.0)	0.06
≤ 68 years	442 (79.4)	407 (72.5)	0.15	397 (77.7)	383 (74.9)	0.06
Current/former smoker						
Yes	174 (31.2)	151 (26.9)	0.09	155 (30.3)	146 (28.6)	0.03
No	383 (68.8)	410 (73.1)	0.09	356 (69.7)	365 (71.3)	0.03
Alcohol history						
Yes	114 (20.5)	96 (17.1)	0.08	99 (19.4)	96 (18.8)	0.01
No	443 (79.5)	465 (82.9)	0.08	412 (80.6)	415 (81.2)	0.01
BMI						
≥ 25 kg/m^2^	159 (28.5)	132 (22.7)	0.22	127 (24.9)	109 (21.3)	0.08
< 25 kg/m^2^	398 (71.5)	429 (76.5)	0.22	384 (75.1)	402 (78.7)	0.08
ASA score						
Grade I	237 (42.5)	265 (47.2)	0.09	218 (42.7)	222 (43.4)	0.01
Grade II or III	320 (57.5)	296 (52.8)	0.09	293 (57.3)	289 (56.6)	0.01
Comorbidity condition						
Age‐adjusted CCI > 3	323 (58.0)	319 (56.9)	0.02	304 (59.5)	280 (54.8)	0.09
Coronary artery disease	60 (10.8)	39 (7.0)	0.15	45 (8.8)	33 (6.5)	0.06
Cerebrovascular disease	55 (9.9)	50 (8.9)	0.03	53 (10.4)	47 (9.2)	0.04
Diabetes mellitus	47 (8.4)	43 (7.7)	0.02	41 (8.0)	34 (6.7)	0.05
COPD/emphysema	41 (7.4)	54 (9.6)	0.07	40 (7.8)	50 (9.8)	0.06
Max‐diameter of lesion						
> 1.8 cm	327 (58.7)	382 (68.1)	0.20	317 (62.0)	338 (66.1)	0.08
≤ 1.8 cm	230 (41.3)	179 (31.9)	0.20	194 (38.0)	173 (33.9)	0.08
Differentiation						
Well	157 (28.2)	191 (34.0)	0.12	154 (30.1)	157 (30.7)	0.01
Moderate or poor	400 (71.8)	370 (65.9)	0.12	357 (69.9)	354 (69.3)	0.01
LVI/PNI						
Yes	9 (1.6)	3 (0.5)	0.14	3 (0.6)	3 (0.6)	0
No	548 (98.4)	558 (99.5)	0.14	508 (99.4)	508 (99.4)	0
T staging						
T1a‐m1	151 (27.8)	344 (61.3)	0.70	137 (26.8)	316 (61.8)	0.35
T1a‐m2–T1a‐m3	201 (36.1)	155 (27.6)	0.18	186 (36.4)	139 (27.2)	0.09
T1b	205 (36.8)	62 (11.1)	0.82	188 (36.8)	56 (11.0)	0.26

Abbreviations: ASA, American Society of Anesthesiologists; BMI, body mass index; CCI, Charlson comorbidity index; COPD, chronic obstructive pulmonary disease; ESD, endoscopic submucosal dissection; LVI/PNI, lymphvascular/perineural invasion; T1a‐m1, intra‐epithelium; T1a‐m2, lamina propria; T1a‐m3, mucosa muscularis; T1b, submucosa; TLME, thoraco‐laparoscopic McKeown esophagectomy.

### 3.2 Operational Parameters and Complications

As showed in Table S2, there was no 30‐ or 90‐day mortality. One hundred and ten patients (19.6%) had piecemeal resection and 92 (16.4%) had R1‐resection in the ESD group, respectively. Duration of procedure (mean, 201 vs. 103 min, *p* < 0.001), bleeding volume (mean, 169 vs. 58 mL, *p* < 0.001), and postoperative length of stay (mean, 16 vs. 12 days, *p* < 0.001) favored ESD. The ESD group also had fewer esophageal fistulas (6.2% vs. 1.3%, *p* < 0.001). However, more ESD patients needed a second endoscopic procedure for delayed bleeding (0.2% vs. 2.3%, *p* = 0.001).

After PS‐matching, duration of procedures (mean, 194 vs. 101 min, *p <* 0.001), bleeding volume (mean, 170 vs. 62 mL, *p <* 0.001), and postoperative length of stay (mean, 16 vs. 12 days, *p <* 0.001) still favored ESD as expected. We found better outcomes in the ESD group with respect to esophageal fistula (6.7% vs. 1.2%, *p <* 0.001) and unplanned intubation (1.4% vs. 0.4%, *p* < 0.001). Delayed bleeding was more likely to occur in the ESD group than the TLME group (0.2% vs. 2.5%, *p <* 0.001).

### 3.3 Long‐Term Complications

In comparison of long‐term complications in Table S2 (each complication is treated as a separate binary variable), the TLME group had more pneumonia (26.7% vs. 13.0%, *p <* 0.001), gastric retention (4.7% vs. 2.0%, *p =* 0.012), and severe reflux (7.7% vs. 4.3%, *p =* 0.016). However, more patients in the ESD group suffered dysphagia (31.8% vs. 27.7%, *p =* 0.009) and needed endoscopic treatment (14.7% vs. 22.4%, *p =* 0.001). The result remained similar after PS matching; more patients in the TLME group had pneumonia (27.2% vs. 13.1%, *p <* 0.001), severe reflux (7.4% vs. 4.3%, *p <* 0.001), and gastric retention (4.7% vs. 2.0%, *p <* 0.001). Dysphagia (20.2% vs. 28.6%, *p <* 0.001) still occurred more in the ESD group and needed endoscopic treatment (14.3% vs. 23.1%, *p =* 0.001).

### 3.4 Survival

**FIGURE 2:**
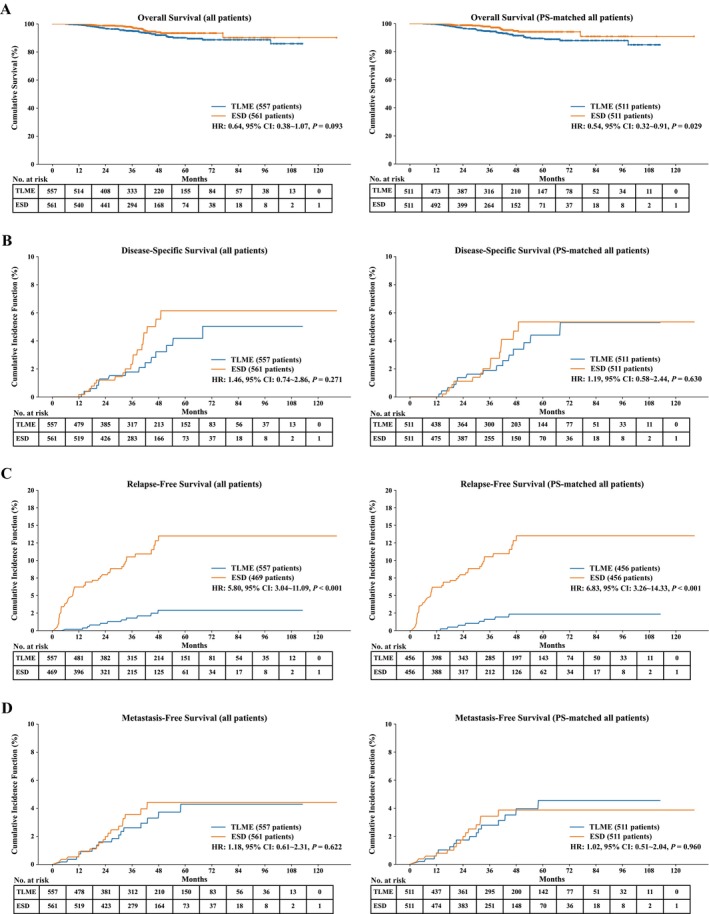
Survival curve and Kaplan–Meier analyses of cT1N0 ESCC patients undergoing TLME and ESD before (left panel) and after (right panel) PS‐matching. (A) Overall survival; (B) disease‐specific survival; (C) relapse‐free survival; (D) metastasis‐free survival.

**FIGURE 3:**
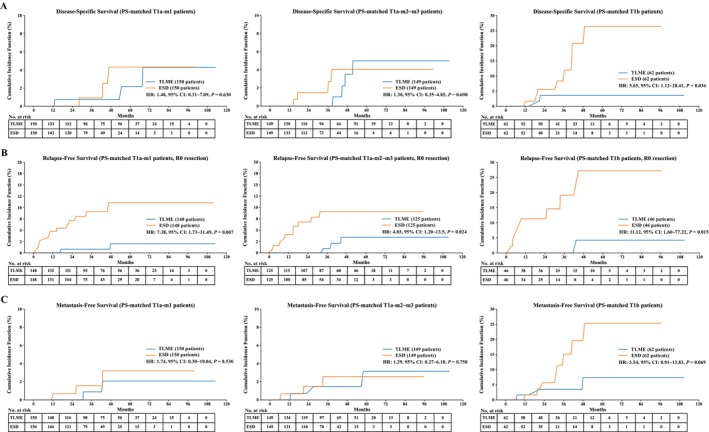
Subgroup survival curve and Kaplan–Meier analyses of cT1N0 ESCC patients undergoing TLME and ESD in PS‐matched cohorts, depending on invasion depth. (A) Disease‐specific survival of patients from T1a‐m1 to T1b subgroup. (B) Relapse‐free survival of patients from T1a‐m1 to T1b subgroup. (C) Metastasis‐free survival of patients from T1a‐m1 to T1b subgroup.

Within a median follow‐up of 38.9 months, 58 patients died. After PS‐matching, all‐cause mortality was higher in the TLME group (38/511 vs. 17/511, 7.4% vs. 3.3%, *p* = 0.004, Table S2). K–M estimation observed a better OS in the ESD group (HR: 0.54, 95% CI: 0.32–0.91, *p* = 0.029, Figure 2A, right panel). Fine and Gray estimation of the DSS curve showed comparable results both in the unmatched (*p* = 0.271) and matched groups (*p* = 0.630) (Figure 2B). However, subgroup analysis demonstrated that T1b cancer had significantly better DSS after being treated by TLME in both unmatched (HR: 2.38, 95% CI: 1.04–5.48, *p* = 0.042, Figure S2A, right panel) and matched T1b subgroups (HR: 5.65, 95% CI: 1.12–28.41, *p* = 0.036, Figure 3A, right panel).

Among patients with R0 resection, 69 experienced cancer relapse. The ESD group was associated with a higher risk of cancer relapse in unmatched (HR: 5.80, 95% CI: 3.04–11.09, *p* < 0.001) and PS‐matched cohort (HR: 6.83, 95% CI: 3.26–14.33, *p* < 0.001) (Figure 2C). Subgroup Fine and Gray analyses showed better RFS in the TLME group in both unmatched (Figure S2B) and matched subgroups (Figure 3B). Metastasis occurred in 34 patients, with comparable MFS in all patients (Table S2, Figure 2D) and T1a subgroup (Figure 3C, Figure S2C). However, MFS showed better results after TLME in T1b cancer (HR: 2.88, 95% CI: 1.14–7.24, *p* = 0.025, Figure S2C), and PS‐matched comparative analyses showed similar results (HR: 3.54, 95% CI: 0.91–13.83, *p* = 0.069) (Figure 3C, Figure S2C).

Survival outcome between R1 and R0 resection among ESD group was depicted in Figure S3. R1 resection patients had worse OS (HR: 5.89, 95% CI: 1.66–20.94, *p* = 0.006), worse DSS (HR: 3.67, 95% CI: 1.45–9.28, *p* = 0.006), and worse MFS (HR: 3.60, 95% CI: 1.42–9.15, *p* = 0.007). However, RFS showed no difference (HR: 0.98, 95% CI: 0.48–1.99, *p* = 0.960).

## 4 Discussion

This is the largest study using real‐world data to compare TLME and ESD for cT1N0 stage ESCC. Analyzing over 1000 patients, ESD showed better OS overall, but not significantly in subgroup analyses compared to TLME. DSS, MFS, and RFS were worse after ESD for T1b cancers invading the submucosa. T1b was an independent predictor of worse OS, DSS, and MFS.

Recent data shows all‐cause mortality for clinical T1 stage esophagectomy ranges from 10.9% to 14.1% and disease‐specific mortality from 7.4% to 13.4% [17, 18]. Our series showed 2.5% all‐cause and 6.8% disease‐specific mortality with a similar follow‐up period. This better result may be due to the inclusion of open procedures in other studies, which have higher mortality than minimally invasive esophagectomy [19]. We also excluded patients with pathologically confirmed LNM, which may have led to better survival outcomes.

ESD was reported to be of greater curative potential than other endoscopic procedure [20, 21]. However, R1 resection occurred in 19.2% of cases and was an independent risk factor for relapse and metastasis in our study (Table S3). Among 104 patients with R1 resection, only 12 patients who underwent endoscopic mucosal resection accepted immediate esophagectomy (crossed over to TLME). The other 92 patients who received ESD under surveillance without additional surgery had worse OS, DSS, and MFS than those with R0 resection (Figure S2), highlighting the importance of additional treatment after non‐curative ESD.

Therapeutic strategies following non‐curative ESD for early‐stage EC remain controversial. Li et al. [22] and Liu et al. [23] reported that additional surgery after non‐curative ESD resulted in better survival than no further treatment. Chemoradiotherapy (CRT) as an esophagus‐preserving option showed comparable OS and DSS to additional surgery but had a higher incidence of cancer relapse [24, 25]. Therefore, esophagectomy seems to be a reasonable remedy for early ESCC after non‐curative ER. However, patients and families often prefer CRT or a wait‐and‐see approach after R1 resection. This highlights the need to evaluate nonsurgical treatments for safety and survival, and to identify factors for disease progression after non‐curative ER. Integrated information would help balance patient decisions and offer the most appropriate treatment.

A common debate of ER for early esophageal cancer centers on insufficient curability regarding untreated lymph nodes, which might cause cancer relapse or disease progression in the long term. Zhang et al. [17] reported that superficial ESCC patients had a lower risk of node metastasis after esophagectomy than ESD. We found the prognostic benefit of TLME for cancer metastasis was confined to the T1b subgroup. Histopathology found 36 pre‐diagnosed T1N0 ESCC cases had metastasized nodes after esophagectomy, indicating a nonzero risk of missed LNM in T1b cancers after ESD without lymphadenectomy. Thus, T1b cancers at high risk for LNM might benefit from surgery with lymphadenectomy. Accurate T staging and understanding LNM patterns are crucial for identifying candidates for ER or surgery. However, EUS and PET/CT are unreliable for assessing LNM in early‐stage esophageal cancer. EUS accuracy for T staging in early cancers is questioned, affecting LNM estimation based on invasion depth [25, 26].

ESD's superiority over esophagectomy in perioperative safety is evident from procedure parameters and post‐treatment complications, impacting 90‐day mortality risk and quality of life (QOL). This is supported by the development of less invasive procedures with enhanced recovery after surgery [27]. The safety of surgical procedure and perioperative nursing management after esophagectomy has been greatly improved. Despite the higher fistula rate in the TLME group, the 90‐day mortality of TLME is well controlled in this series. Kamarajah et al. [11] reported a 90‐day mortality of 4.1% (40/964) after esophagectomy for cT1N0 EC based on the National Cancer Database. Though not specified, the work included patients undergoing open procedures which might have led to higher hospital mortality [28]. Stricture, a major detriment to QOL after ER and esophagectomy, occurred more in the ESD group (15.4% vs. 21.5% for TLME) but was acceptable compared to previously reported data (6.5%–60% after ESD [29, 30, 31], 0%–63% after esophagectomy [32, 33, 34]).

Limitations should be noted. First, we tried to balance the selection bias by performing PSM and subgroup analysis. However, some potential selection and referral biases still exist due to the retrospective nature of the study. Furthermore, this study only assessed thoraco‐laparoscopic esophagectomy; it is of interest to compare ER with less invasive esophagectomy procedures such as laparo‐mediastinoscopy McKeown esophagectomy [35, 36], or total minimally invasive Ivor‐Lewis esophagectomy [37] for early‐stage EC. Although we use EUS to determine the depth of tumor infiltration in each patient preoperatively, the results were not always completely accurate, which led to some T1b patients undergoing ESD. However, we believe there is currently no absolutely accurate method to differentiate between T1a‐m3 and T1b. Therefore, this study reflects the actual clinical situation. Finally, during follow‐up, most patients were unable to accurately recall the onset of long‐term complications. As a result, we were unable to report long‐term complications using a linearized risk approach, which may overlook the impact of time on these outcomes.

In summary, TLME for clinical T1N0 ESCC is superior to ESD for cancer relapse in all sub‐T1 stages and provides better DSS and MFS in the T1b stage. However, ESD had better OS.

## Author Contributions

**Ping Yuan:** conceptualization, methodology, validation, formal analysis, investigation, writing – review and editing, supervision, project administration, and funding acquisition. **Zhenhao Huang:** conceptualization, methodology, software, formal analysis, investigation, data curation, writing – original draft, and visualization. **Yunhai Yang** and **Feichao Bao:** conceptualization, validation, and writing – review and editing. **Xiangnan Li:** validation, investigation, resources, and data curation. **Lidong Chen** and **Hongtao Wen:** investigation, resources and data curation. **Jiajing Li** and **Donglei Liu:** validation, investigation, and data curation. **Feng Li:** data curation. **Shanfeng Zhang:** methodology and investigation. **Yu Qi:** conceptualization, methodology, resources, supervision, project administration, and funding acquisition.

## Conflicts of Interest

The authors declare no conflicts of interest.

## Supporting information


Figure S1.
Figure S2.
Figure S3.
Figure S4.

Table S1.
Table S2.
Table S3.
Table S4.

## Data Availability

The data underlying this article were accessed from our prospectively assembled database (https://zzutsd.com) and institutionally maintained medical record system. The derived data generated in this research will be shared on reasonable request to the corresponding author.
