# Supplementary material for: Survival Outcome of Thoraco‐Laparoscopic McKeown Esophagectomy Versus Endoscopic Submucosal Dissection for Early‐Stage Esophageal Squamous Cell Carcinoma: A Propensity Score‐Matched Analysis
Source: Thorac Cancer. 2025 May 5;16(9):e70064. doi: 10.1111/1759-7714.70064 (PMC12052754; doi:10.1111/1759-7714.70064)
Supplement: Supplementary file 1 — Figure S1. Figure S2. Figure S3. Figure S4. [file TCA-16-e70064-s001.docx]

**Supplementary Figure**

**Title: Survival outcome of thoraco-laparoscopic McKeown esophagectomy versus endoscopic submucosal dissection for early stage esophageal squamous cell carcinoma depending on invasion depth: A propensity-score matched analysis**

**CONTENT**

1. **Supplementary** **Figure 1:** Covariance balance plot depicted by absolute standardized difference (ASD) before and after propensity-score matching (PS-matching) for patients undergoing TLME and ESD. The triangle and square symbol represent corresponding ASD before and after PS-matching, respectively. A symbol appearing in the green area means that the relavant ASD is less than 0.1 and denoted a negligible difference between the 2 groups.

A and B: plot of the whole cases and patients underwent R0 resection.

C and D: plot of T1a-m1 subgroup patients and T1a-m1 subgroup underwent R0 resection

E and F: plot of T1a-m2~m3 subgroup patients and T1a-m2~m3 subgroup underwent R0 resection

G and H: plot of T1b subgroup patients and Tb subgroup underwent R0 resection

1. **Supplementary** **Figure 2:** Subgroup survival curve and Kaplan-Meier (K-M) analyses of cT1N0 ESCC patients undergoing TLME and ESD before PS-matching, depending on invasion depth.

A: Disease-specific survival of patients from T1a-m1 to T1b subgroup

B: Relapse-free survival of patients from T1a-m1 to T1b subgroup

C: Metastasis-free survival of patients from T1a-m1 to T1b subgroup

1. **Supplementary Figure 3:** Survival curve and Kaplan-Meier (K-M) analyses of cT1N0 ESCC patients received R0-resection and R1-resection after ESD treatment.

A: Overall survival curve

B: Disease-specific survival

C: Relapse-free survival

D: Metastasis-free survival

1. **Supplementary Figure 4:** Density plots of different groups before and after the PS-matching showed the overlap.

A: all patients

B: T1a-m1 patients

C: T1a-m2~m3 patients

D: T1b patients


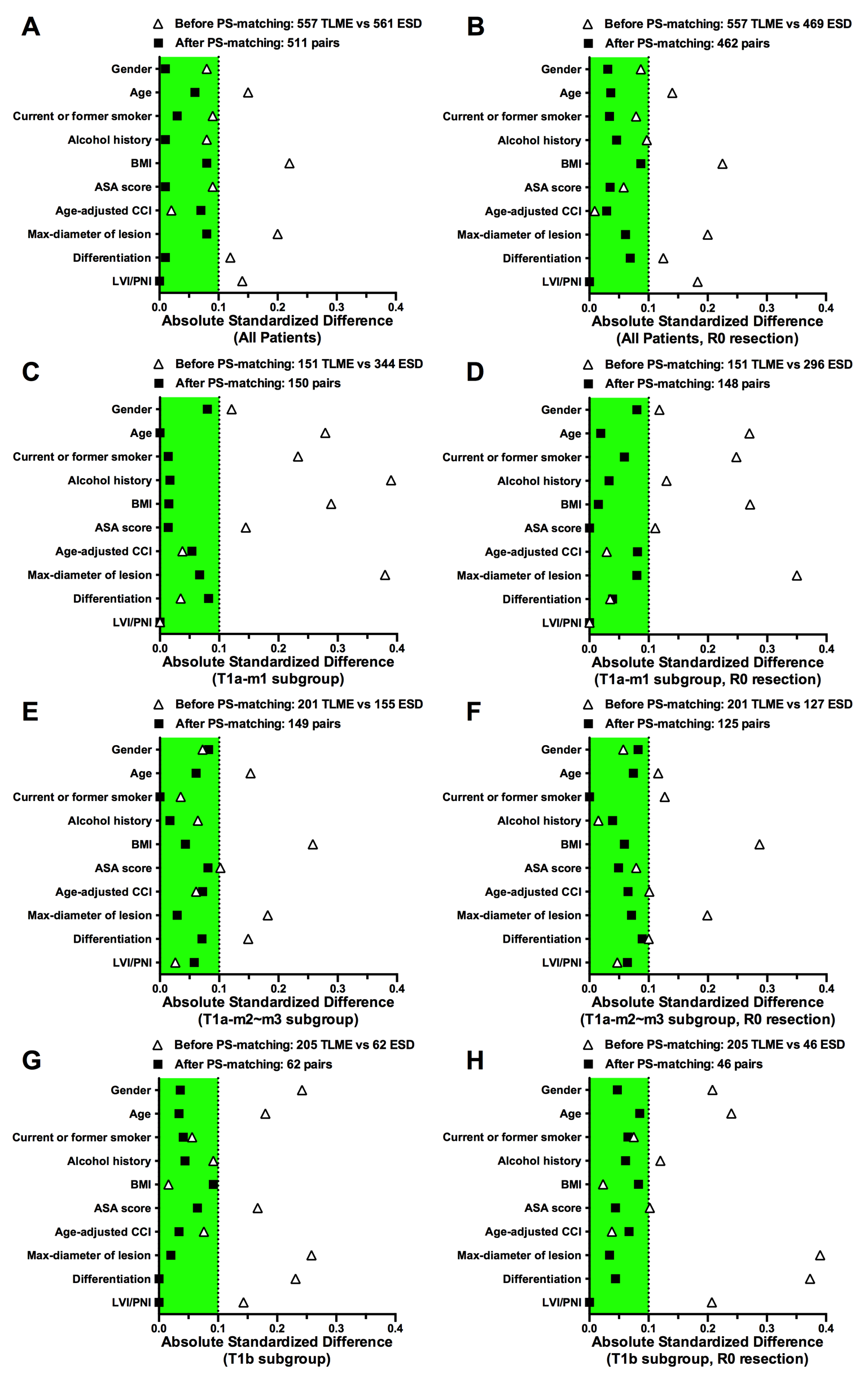


**Supplementary** **Figure 1:** Covariance balance plot depicted by absolute standardized difference (ASD) before and after propensity-score matching (PS-matching) for patients undergoing TLME and ESD. The triangle and square symbol represent corresponding ASD before and after PS-matching, respectively. A symbol appearing in the green area means that the relavant ASD is less than 0.1 and denoted a negligible difference between the 2 groups.

A and B: plot of the whole cases and patients underwent R0 resection.

C and D: plot of T1a-m1 subgroup patients and T1a-m1 subgroup underwent R0 resection

E and F: plot of T1a-m2~m3 subgroup patients and T1a-m2~m3 subgroup underwent R0 resection

G and H: plot of T1b subgroup patients and Tb subgroup underwent R0 resection





**Supplementary** **Figure 2:** Subgroup survival curve and Kaplan-Meier (K-M) analyses of cT1N0 ESCC patients undergoing TLME and ESD before PS-matching, depending on invasion depth.

A: Disease-specific survival of patients from T1a-m1 to T1b subgroup

B: Relapse-free survival of patients from T1a-m1 to T1b subgroup

C: Metastasis-free survival of patients from T1a-m1 to T1b subgroup


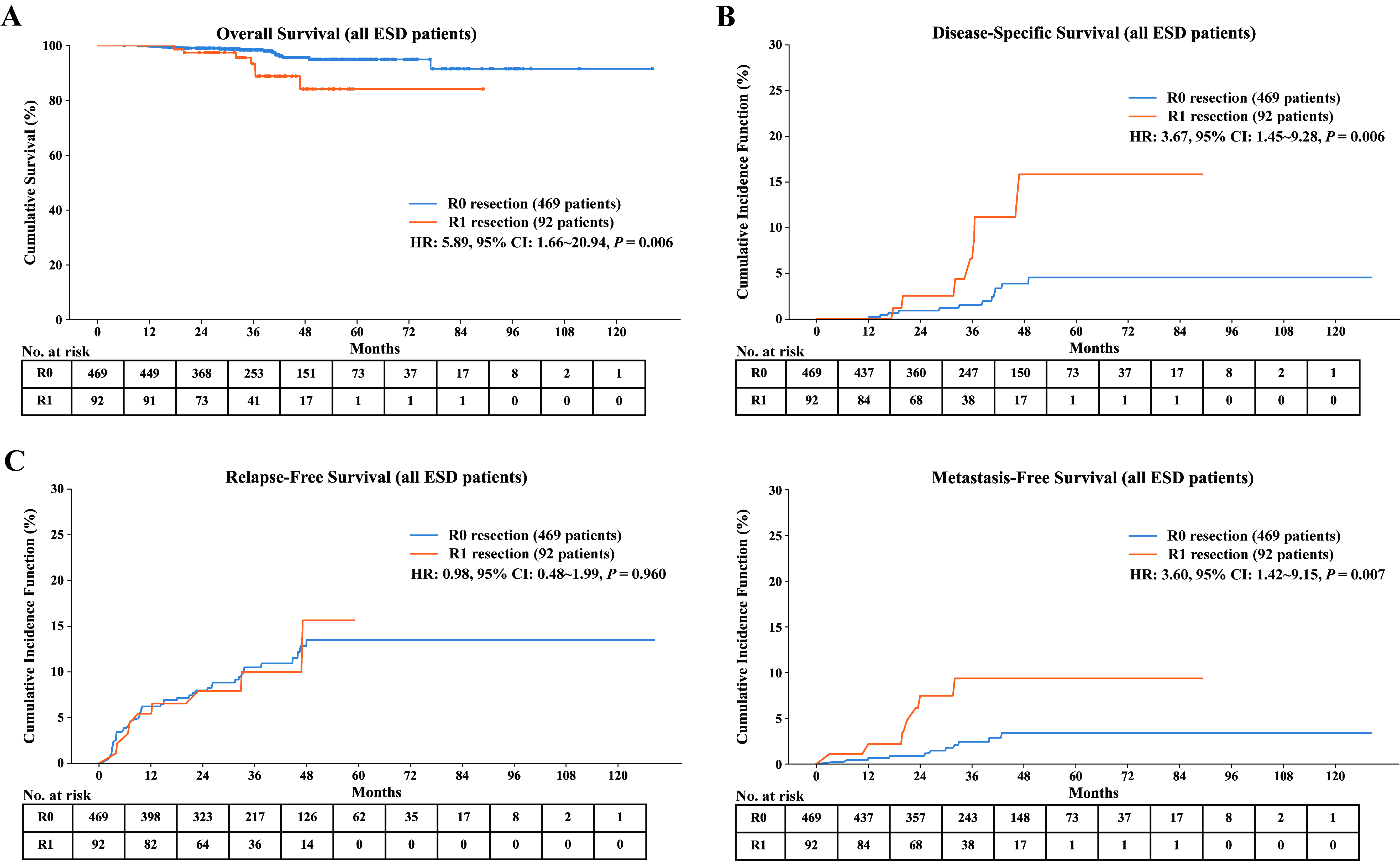


**Supplementary Figure 3:** Survival curve and result of Kaplan-Meier (K-M) analyses of cT1N0 ESCC patients underwent R0-resection and R1-resection in ESD group.

A: Overall survival

B: Disease-specific survival

C: Relapse-free survival

D: Metastasis-free survival


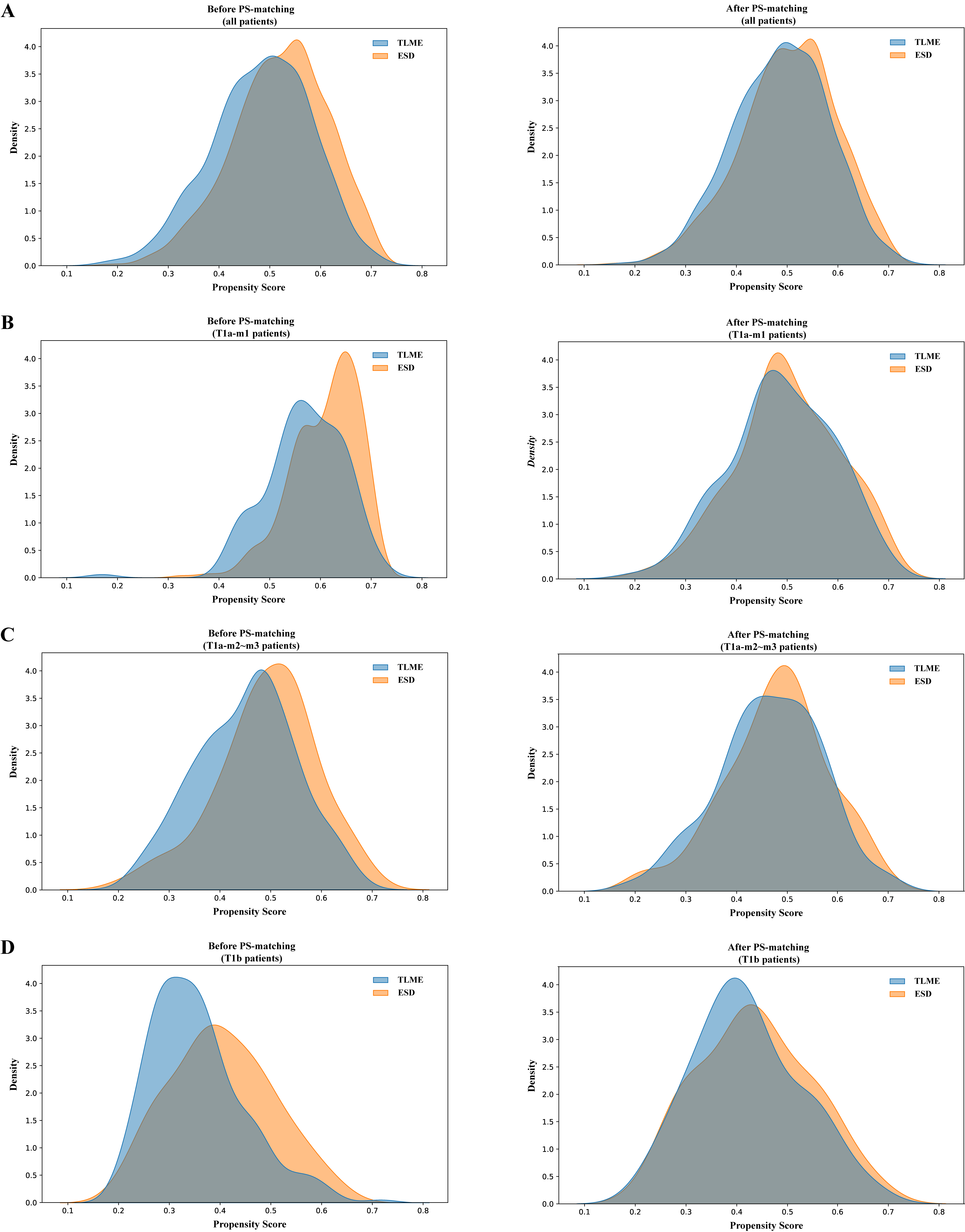


**Supplementary Figure 4:** Density plots of different groups before and after the PS-matching showed the overlap.

A: all patients

B: T1a-m1 patients

C: T1a-m2~m3 patients

D: T1b patients
