# Supplementary material for: Survival Outcome of Thoraco‐Laparoscopic McKeown Esophagectomy Versus Endoscopic Submucosal Dissection for Early‐Stage Esophageal Squamous Cell Carcinoma: A Propensity Score‐Matched Analysis
Source: Thorac Cancer. 2025 May 5;16(9):e70064. doi: 10.1111/1759-7714.70064 (PMC12052754; doi:10.1111/1759-7714.70064)
Supplement: Supplementary file 2 — Table S1. Table S2. Table S3. Table S4. [file TCA-16-e70064-s002.docx]

**Supplementary Table**

**Title: Survival outcome of thoraco-laparoscopic McKeown esophagectomy versus endoscopic submucosal dissection for early stage esophageal squamous cell carcinoma depending on invasion depth: A propensity-score matched analysis**

**CONTENT**

1. **Supplementary Table 1:** Demographic, Clinical Characteristics of subgroup Patients with calculated absolute standardized difference (ASD) before propensity-score matching (PS-matching)
2. **Supplementary Table 2:** Clinical outcomes of all patients and propensity score (PS)-matched group undergoing thoraco-laparoscopic McKeown esophagectomy (TLME) or endoscopic submucosal dissection (ESD) for cT1N0 ESCC
3. **Supplementary Table 3:** Uni-variate and multi-variate Cox-regression of factors associated with survival for cT1N0 ESCC (number in dataframe = 1118)
4. **Supplementary Table 4:** Demographic, Clinical Characteristics of subgroup Patients with calculated absolute standardized difference (ASD) after propensity-score matching (PS-matching)

| **Supplementary table 1. Demographic and clinical characteristics of subgroup patients with calculated absolute standardized difference (ASD) before propensity-score matching (PS-matching)** | | | | | | | | | |
| --- | --- | --- | --- | --- | --- | --- | --- | --- | --- |
| Factor (no. %) | T1a-m1 | |  | T1a-m2~m3 | |  | T1b | |  |
|  | 151 TLME | 344 ESD | ASD | 201 TLME | 155 ESD | ASD | 205 TLME | 62 ESD | ASD |
| Gender |  |  |  |  |  |  |  |  |  |
| Male | 89 (58.9) | 182 (52.9) | 0.12 | 129 (64.2) | 94 (60.6) | 0.07 | 123 (60.0) | 44 (71.0) | 0.24 |
| Female | 62 (41.1) | 162 (47.1) | 0.12 | 72 (35.8) | 61 (39.4) | 0.07 | 82 (40.0) | 18 (29.0) | 0.24 |
| Age |  |  |  |  |  |  |  |  |  |
| >68 years | 21 (13.9) | 90 (26.2) | 0.27 | 42 (20.9) | 43 (27.7) | 0.15 | 52 (25.4) | 21 (33.9) | 0.18 |
| ≤68 years | 130 (86.1) | 254 (73.8) | 0.27 | 159 (79.1) | 112 (72.3) | 0.15 | 153 (74.6) | 41 (66.1) | 0.18 |
| Current/former smoker |  |  |  |  |  |  |  |  |  |
| Yes | 51 (33.8) | 82 (32.8) | 0.23 | 59 (29.4) | 48 (31.0) | 0.04 | 64 (31.2) | 21 (33.9) | 0.06 |
| No | 100 (66.2) | 262 (67.2) | 0.23 | 142 (70.6) | 107 (69.0) | 0.04 | 141 (68.8) | 41 (66.1) | 0.06 |
| Alcohol history |  |  |  |  |  |  |  |  |  |
| Yes | 30 (19.9) | 56 (16.3) | 0.40 | 44 (21.9) | 30 (19.4) | 0.06 | 40 (19.5) | 10 (16.1) | 0.09 |
| No | 121 (80.1) | 288 (83.7) | 0.40 | 157 (78.1) | 125 (80.6) | 0.06 | 165 (80.5) | 52 (83.9) | 0.09 |
| BMI |  |  |  |  |  |  |  |  |  |
| ≥25 kg/m2 | 44 (29.1) | 63 (18.3) | 0.29 | 64 (31.8) | 33 (21.3) | 0.26 | 51 (24.9) | 15 (24.2) | 0.02 |
| ＜25 kg/m2 | 107 (70.9) | 281 (82.0) | 0.29 | 137 (68.2) | 122 (78.7) | 0.26 | 154 (75.1) | 47 (75.8) | 0.02 |
| ASA score |  |  |  |  |  |  |  |  |  |
| Grade I | 93 (61.6) | 187 (54.4) | 0.15 | 114 (56.7) | 80 (51.6) | 0.10 | 158 (77.1) | 41 (66.1) | 0.17 |
| Grade II or III | 58 (38.4) | 157 (45.6) | 0.15 | 87 (43.3) | 75 (48.4) | 0.10 | 47 (22.9) | 21 (33.9) | 0.17 |
| Comorbidity condition |  |  |  |  |  |  |  |  |  |
| Age-adjusted CCI >3 | 81 (53.6) | 191 (55.5) | 0.04 | 112 (55.2) | 91 (58.7) | 0.06 | 130 (63.4) | 37 (59.7) | 0.08 |
| Coronary artery disease | 10 (6.6) | 22 (6.4) | 0.01 | 27 (13.4) | 9 (5.8) | 0.33 | 23 (11.2) | 8 (12.9) | 0.05 |
| Cerebrovascular disease | 9 (6.0) | 28 (8.1) | 0.08 | 21 (10.4) | 16 (10.3) | 0.01 | 25 (12.2) | 6 (9.7) | 0.09 |
| Diabetes mellitus | 12 (7.9) | 29 (8.4) | 0.02 | 16 (8.0) | 7 (4.5) | 0.17 | 19 (9.3) | 7 (11.3) | 0.06 |
| COPD/emphysema | 8 (5.3) | 33 (9.6) | 0.15 | 16 (8.0) | 12 (7.7) | 0.01 | 17 (8.3) | 9 (14.5) | 0.18 |
| Max-diameter of lesion |  |  |  |  |  |  |  |  |  |
| >1.8 cm | 72 (47.7) | 226 (65.7) | 0.38 | 126 (62.7) | 110 (71.0) | 0.18 | 129 (62.9) | 46 (74.2) | 0.26 |
| ≤1.8cm | 79 (52.3) | 118 (34.3) | 0.38 | 75 (37.3) | 45 (29.0) | 0.18 | 76 (37.1) | 16 (25.8) | 0.26 |
| Differentiation |  |  |  |  |  |  |  |  |  |
| Well | 98 (64.9) | 229 (66.6) | 0.04 | 144 (71.6) | 100 (64.5) | 0.15 | 158 (77.1) | 41 (76.1) | 0.23 |
| Moderate or poor | 53 (35.1) | 115 (33.4) | 0.04 | 57 (28.4) | 55 (35.5) | 0.15 | 47 (22.9) | 21 (33.9) | 0.23 |
| LVI/PNI |  |  |  |  |  |  |  |  |  |
| Yes | 0 (0.0) | 0 (0.0) | 0 | 2 (1.0) | 2 (1.3) | 0.03 | 7 (3.4) | 1 (1.6) | 0.14 |
| No | 151 (100.0) | 344 (100.0) | 0 | 199 (99.0) | 153 (98.7) | 0.03 | 198 (96.6) | 61 (98.4) | 0.14 |
| TLME: Thoraco-laparoscopic McKeown Esophagectomy; ESD: Endoscopic submucosal dissection; BMI: Body mass index; ASA: American Society of Anesthesiologists; CCI: Charlson comorbidity index; COPD: chronic obstructive pulmonary disease; LVI/PNI: lymphvascular/perineural invasion; T1a-m1: intra-epithelium; T1a-m2: lamina propria; T1a-m3: mucosa muscularis; T1b: submucosa; | | | | | | | | | |

| **Supplementary table 2. Clinical outcomes of all patients and propensity score (PS)-matched group undergoing thoraco-laparoscopic McKeown esophagectomy (TLME) or endoscopic submucosal dissection (ESD) for cT1N0 ESCC** | | | | | | |
| --- | --- | --- | --- | --- | --- | --- |
| Factor | All patients | |  | PS-matched patients | |  |
|  | 557 TLME | 561 ESD | *P* | 511 TLME | 511 ESD | *P* |
| Duration of procedure (min, MSD) | 201 ± 83 | 103 ± 82 | <0.001 | 194 ± 72 | 101 ± 80 | <0.001 |
| Bleeding volume (ml, MSD) | 169 ± 96 | 58 ± 15 | <0.001 | 170 ± 73 | 62 ± 20 | <0.001 |
| No. Lymph node dissected (MSD) | 9 ± 1 | 0 (0) | NA | 8 ± 1 | 0 (0) | NA |
| Piecemeal resection (no. %) | 0 (0) | 110 (19.6) | NA | 0 (0) | 98 (19.2) | NA |
| R1 resection | 0 (0) | 92 (16.4) | NA | 0 (0) | 83 (16.2) | NA |
| Adverse Event (no. %) |  |  |  |  |  |  |
| Delayed bleeding | 1 (0.2) | 13 (2.3) | 0.001 | 1 (0.2) | 13 (2.5) | <0.001 |
| Fistula | 36 (6.2) | 7 (1.3) | <0.001 | 34 (6.7) | 6 (1.2) | <0.001 |
| Unplanned intubation | 7 (1.3) | 2 (0.4) | 0.093 | 7 (1.4) | 2 (0.4) | <0.001 |
| Postoperative length of stay (days, MSD) | 16 ± 8 | 12 ± 8 | <0.001 | 17 ± 9 | 12 ± 5 | <0.001 |
| 90-day mortality (no. %) | 0 (0) | 1 (0.2) | NA | 0 (0) | 0 (0) | NA |
| Long-term complication |  |  |  |  |  |  |
| Pneumonia | 149 (26.7) | 73 (13.0) | <0.001 | 139 (27.2) | 67 (13.1) | <0.001 |
| Severe reflux^a^ | 43 (7.7) | 24 (4.3) | 0.016 | 38 (7.4) | 22 (4.3) | <0.001 |
| Gastric retention | 26 (4.7) | 11 (2.0) | 0.012 | 24 (4.7) | 10 (2.0) | <0.001 |
| Vocal cord dysfunction | 12 (2.2) | 4 (0.7) | 0.042 | 11 (2.2) | 4 (0.8) | <0.001 |
| Dysphagia | 117 (21.0) | 155 (27.8) | 0.009 | 103 (20.2) | 146 (28.6) | <0.001 |
| Subsequent treatment |  |  |  |  |  |  |
| Dilation or stent placement for stricture | 82 (14.7) | 126 (22.4) | 0.001 | 73 (14.3) | 118 (23.1) | 0.001 |
| Endoscopic treatment for cancer relapse | 2 (0.2) | 50 (8.9) | <0.001 | 1 (0.2) | 45 (8.8) | <0.001 |
| Chemotherapy | 21 (3.8) | 33 (5.9) | 0.118 | 21 (4.1) | 33 (6.5) | 0.128 |
| Cancer relapse | 11 (2.0) | 58 (10.3) | <0.001 | 9 (1.8) | 53 (10.4) | <0.001 |
| Cancer metastasis | 16 (2.9) | 18 (3.2) | 0.744 | 16 (3.1) | 15 (2.9) | 0.855 |
| All-cause mortality | 38 (6.8) | 20 (3.6) | 0.014 | 38 (7.4) | 17 (3.3) | 0.004 |
| Disease-specific mortality | 15 (2.7) | 18 (3.2) | 0.611 | 15 (2.9) | 15 (2.9) | NA |
| MSD: Mean with standard deviation; ^a^Severe reflux: reflux with heartburn (≥ 3 times/day)  Gastric retention: characterized by symptoms suggesting retention of food in the stomach with objective evidence of delayed gastric emptying in the absence of mechanical obstruction in the gastric outflow.  The categorical variables form Adverse Event and Long-term complication were tested using the Chi-squared test before matching and the McNemar test after matching. | | | | | | |

| **Supplementary table 3. Uni-variate and multi-variate Cox-regression of factors associated with survival for cT1N0 ESCC (number in dataframe = 1118)** | | | | | | | | |
| --- | --- | --- | --- | --- | --- | --- | --- | --- |
| Variables | Overall survival | | Disease-specific survival | | Relapse free survival | | Metastasis free survival | |
|  | Uni-variate HR (95% CI),  *P* value | Multi-variate HR (95% CI),  *P* value | Uni-variate HR (95% CI),  *P* value | Multi-variate HR (95% CI)*,*  *P* value | Uni-variate HR (95% CI),  *P* value | Multi-variate HR (95% CI),  *P* value | Uni-variate HR (95% CI),  *P* value | Multi-variate HR (95% CI),  P value |
| Age > 68 years,  ≤68 years as Ref. | 2.00 (1.16-3.45), 0.012 | 1.53 (0.84~2.79), 0.166 | 2.14 (1.05~4.37), 0.036 | 3.35 (1.37~8.16), 0.008 | 1.30 (0.77~2.20), 0.334 |  | 1.45 (0.69~3.03), 0.325 |  |
| Male Gender,  female as Ref. | 2.13 (1.18~3.82), 0.012 | 2.10 (1.16~3.81), 0.014 | 3.33 (1.37~8.07), 0.008 | 2.12 (1.03~4.35), 0.041 | 0.97 (0.60~1.57)  0.915 |  | 1.86 (0.87~4.01), 0.111 |  |
| Current/former smoker, never as Ref. | 1.55 (0.92~2.62), 0.103 |  | 1.53 (0.76~3.08), 0.233 |  | 1.37 (0.84~2.24),  0.205 |  | 1.30 (0.65~2.63), 0.461 |  |
| Alcohol history,  none as Ref. | 1.08 (0.57~2.04), 0.811 |  | 1.31 (0.59~2.90), 0.509 |  | 1.50 (0.88~2.57),  0.139 |  | 1.30 (0.59~2.87), 0.515 |  |
| Age-adjusted CCI >3,  0~2 as Ref. | 1.98 (1.05~3.75), 0.035 | 1.54 (0.78~3.04), 0.211 | 1.62 (0.77~3.43), 0.207 |  | 1.16 (0.71~1.91),  0.560 |  | 2.27 (0.85~6.03), 0.101 |  |
| BMI, ≥25 kg/m^2^,  < 24 as Ref. | 0.79 (0.42~1.49), 0.471 |  | 0.68 (0.28~1.64), 0.385 |  | 0.80 (0.44~1.44),  0.454 |  | 0.54 (0.21~1.38), 0.197 |  |
| ASA Grade Ⅱ and Ⅲ, Grade I as Ref. | 1.97 (1.17-3.32), 0.011 | 1.67 (0.96~2.90), 0.068 | 1.78 (0.89~3.54), 0.101 |  | 1.36 (0.85~2.18),  0.201 |  | 1.07 (0.54~2.11), 0.849 |  |
| Lesion max diameter >1.8 cm, ≤ 1.7 cm as Ref. | 1.00 (0.59~1.71), 1.000 |  | 1.28 (0.61~2.70), 0.511 |  | 1.21 (0.73~2.01),  0.462 |  | 0.88 (0.44~1.76), 0.717 |  |
| Poor Differentiation, Moderate and well as Ref. | 0.85 (0.55~1.63), 0.949 |  | 1.05 (0.54~2.03), 0.883 |  | 1.18(0.70~2.00),  0.531 |  | 0.78 (0.37~1.64), 0.516 |  |
| LVI/PNI,  negative as Ref. | 5.41 (1.31~22.27), 0.019 | 1.99 (0.47~8.43), 0.346 | 4.62 (0.63~33.94), 0.133 |  | 1.59 (0.22~11.48),  0.644 |  | 3.94(0.54~28.83), 0.178 |  |
| Ulcerated type of lesion, shallow type as Ref. | 1.02 (0.65~1.78), 0.988 |  | 1.00 (0.58~1.68), 1.000 |  | 1,05 (0.52~2.05) 0.897 |  | 1.00 (0.61~1.85)  1.000 |  |
| The middle and lower segment, upper as Ref. | 0.97 (0.51~1.78), 0.889 |  | 0.95 (0.47~1.65), 0.921 |  | 1.12 (0.65~2.05), 0.977 |  | 0.89 (0.41~1.82) 0.932 |  |
| Invasion depth  T1a-m1 as Ref. |  |  |  |  |  |  |  |  |
| T1a-m2~m3 | 1.17 (0.55~2.50), 0.690 | 0.98 (0.45~2.14), 0.974 | 1.16 (0.56~2.95), 0.741 | 1.07 (0.42~2.72), 0.890 | 0.57 (0.32~1.01),  0.054 | 0.86 ((0.48~1.55), 0.614 | 1.12 (0.42~3.00), 0.824 | 1.16 (0.43~3.12), 0.768 |
| T1b | 4.43 (2.39~8.22), <0.001 | 3.60 (1.84~7.04), 0.001 | 3.22 (1.45~7.17), 0.004 | 3.18 (1.41~7.18), 0.005 | 0.70 (0.38~1.28),  0.246 | 1.56 (0.82~2.99), 0.175 | 4.20 (1.88~9.35), 0.001 | 4.66 (2.08~10.42), 0.001 |
| Piecemeal resection, *en-bloc* resection as Ref. | 0.91 (0.41~2.22), 0.981 |  | 1.44 (0.56~3.72), 0.455 |  | 5.17 (3.15~8.49),  <0.001 | 2.88 (1.70~4.86), <0.001 | 1.81 (0.75~4.37), 0.188 |  |
| R1 resection  R0-resection as Ref. | 1.72 (0.73~4.03), 0.213 |  | 3.26 (1.34~7.97), 0.009 | 3.42 (1.38~8.52), 0.008 | 1.84 (0.9~3.71),  0.089 | 0.99 (0.49~2.03),  0.991 | 3.46 (1.50~7.98), 0.004 | 4.14 (1.78~9.64), 0.001 |
| ESD,  TLME as Ref. | 0.60 (0.35~1.04), 0.068 | 1.16 (0.64~2.08), 0.631 | 1.34 (0.68~2.68), 0.4 |  | 5.75 (3.02~10.96),  <0.001 | 4.73 (2.29~9.78), <0.001 | 1.16 (0.59~2.28), 0.661 |  |
| TLME: Thoraco-laparoscopic McKeown Esophagectomy; ESD: Endoscopic submucosal dissection; HR: Hazard ratio; CI: Confidence interval; CCI: Charlson comorbidity index; BMI: Body mass Index; ASA: American Society of Ansthesiologists; LVI/PNI: lymphvascular/perineural invasion; | | | | | | | | |

| **Supplementary table 4. Demographic and clinical characteristics of subgroup patients with calculated absolute standardized difference (ASD) after propensity-score matching (PS-matching)** | | | | | | | | | |
| --- | --- | --- | --- | --- | --- | --- | --- | --- | --- |
| Factor (no. %) | PS-matched T1a-m1 | |  | PS-matched T1a-m2~3 T1a-m2~m3 | |  | PS-matched T1b | |  |
|  | 150 TLME | 150 ESD | ASD | 149 TLME | 149 ESD | ASD | 62 TLME | 62 ESD | ASD |
| Gender |  |  |  |  |  |  |  |  |  |
| Male | 88 (58.7) | 82 (54.7) | 0.08 | 96 (64.4) | 90 (60.4) | 0.08 | 45 (72.6) | 44 (71.0) | 0.04 |
| Female | 62 (41.3) | 68 (45.3) | 0.08 | 53 (35,6) | 59 (39.6) | 0.08 | 17 (27.4) | 18 (29.0) | 0.04 |
| Age |  |  |  |  |  |  |  |  |  |
| >68 years | 21 (14.0) | 21 (14.0) | 0 | 35 (23.5) | 39 (26.2) | 0.06 | 22 (35.5) | 21 (33.9) | 0.03 |
| ≤68 years | 129 (86.0) | 129 (86.0) | 0 | 114 (76.5) | 110 (73.8) | 0.06 | 40 (74.5) | 41 (66.1) | 0.03 |
| Current/former smoker |  |  |  |  |  |  |  |  |  |
| Yes | 50 (33.3) | 49 (32.7) | 0.01 | 46 (30.9) | 46 (30.9) | 0 | 22 (35.5) | 21 (33.9) | 0.04 |
| No | 100 (66.7) | 101 (67.3) | 0.01 | 103 (69.1) | 103 (69.1) | 0 | 40 (64.5) | 41 (66.1) | 0.04 |
| Alcohol history |  |  |  |  |  |  |  |  |  |
| Yes | 29 (19.3) | 30 (20.0) | 0.02 | 31 (20.8) | 30 (20.1) | 0.02 | 11 (17.7) | 10 (16.1) | 0.04 |
| No | 121 (80.7) | 120 (80.0) | 0.02 | 118 (79.2) | 119 (79.9) | 0.02 | 51 (82.3) | 52 (83.9) | 0.04 |
| BMI |  |  |  |  |  |  |  |  |  |
| ≥25 kg/m2 | 43 (28.7) | 42 (28.0) | 0.02 | 38 (25.5) | 33 (22.1) | 0.04 | 18 (29.0) | 15 (24.2) | 0.09 |
| ＜25 kg/m2 | 107 (71.3) | 108 (72.0) | 0.02 | 111 (74.5) | 116 (77.9) | 0.04 | 44 (71.0) | 47 (75.8) | 0.09 |
| ASA score |  |  |  |  |  |  |  |  |  |
| Grade I | 92 (61.3) | 91 (60.7) | 0.01 | 84 (56.4) | 78 (52.3) | 0.08 | 39 (62.9) | 41 (66.1) | 0.07 |
| Grade II or III | 58 (38.7) | 59 (39.3) | 0.01 | 65 (43.6) | 71 (47.7) | 0.08 | 23 (37.1) | 21 (33.9) | 0.07 |
| Comorbidity condition |  |  |  |  |  |  |  |  |  |
| Age-adjusted CCI >3 | 80 (53.3) | 79 (52.6) | 0.05 | 90 (60.4) | 87 (58.4) | 0.07 | 39 (62.9) | 37 (59.7) | 0.03 |
| Coronary artery disease | 10 (6.7) | 10 (6.7) | 0 | 14 (9.4) | 8 (5.4) | 0.07 | 8 (12.9) | 8 (12.9) | 0 |
| Cerebrovascular disease | 9 (6.0) | 8 (5.3) | 0.03 | 16 (10.7) | 15 (10.1) | 0.02 | 7 (11.2) | 6 (9.7) | 0.08 |
| Diabetes mellitus | 12 (8.0) | 11 (7.3) | 0.03 | 12 (8.1) | 7 (4.7) | 0.07 | 6 (9.6) | 7 (11.3) | 0.09 |
| COPD/emphysema | 8 (5.3) | 12 (8.0) | 0.09 | 15 (10.1) | 12 (8.1) | 0.07 | 8 (12.9) | 9 (14.5) | 0.05 |
| Max-diameter of lesion |  |  |  |  |  |  |  |  |  |
| >1.8 cm | 71 (47.3) | 69 (46.0) | 0.07 | 102 (68.5) | 104 (69.8) | 0.03 | 44 (70.9) | 46 (74.2) | 0.02 |
| ≤1.8cm | 79 (52.3) | 81 (54.0) | 0.07 | 47 (31.5) | 45 (30.2) | 0.03 | 18 (29.1) | 16 (25.8) | 0.02 |
| Differentiation |  |  |  |  |  |  |  |  |  |
| Well | 98 (65.3) | 104 (69.3) | 0.08 | 95 (63.8) | 100 (67.1) | 0.07 | 41 (66.1) | 41 (66.1) | 0 |
| Moderate or poor | 52 (34.7) | 46 (30.6) | 0.08 | 54 (36.2) | 49 (32.9) | 0.07 | 21 (33.9) | 21 (33.9) | 0 |
| LVI/PNI |  |  |  |  |  |  |  |  |  |
| Yes | 0 (0.0) | 0 (0.0) | 0 | 1 (0.7) | 2 (1.3) | 0.06 | 1 (1.6) | 1 (1.6) | 0 |
| No | 150 (100.0) | 150 (100.0) | 0 | 148 (99.3) | 147 (98.7) | 0.06 | 61 (98.4) | 61 (98.4) | 0 |
| TLME: Thoraco-laparoscopic McKeown Esophagectomy; ESD: Endoscopic submucosal dissection; BMI: Body mass index; ASA: American Society of Anesthesiologists; CCI: Charlson comorbidity index; COPD: chronic obstructive pulmonary disease; LVI/PNI: lymphvascular/perineural invasion; T1a-m1: intra-epithelium; T1a-m2: lamina propria; T1a-m3: mucosa muscularis; T1b: submucosa; | | | | | | | | | |
